# Design of a comparative effectiveness randomized controlled trial testing a faith-based Diabetes Prevention Program (WORD DPP) vs. a Pacific culturally adapted Diabetes Prevention Program (PILI DPP) for Marshallese in the United States

**DOI:** 10.1097/MD.0000000000010677

**Published:** 2018-05-11

**Authors:** Pearl Anna McElfish, Christopher R. Long, Joseph Keawe‘aimoku Kaholokula, Nia Aitaoto, Zoran Bursac, Lucy Capelle, Melisa Laelan, Williamina Ioanna Bing, Sheldon Riklon, Brett Rowland, Britni L. Ayers, Ralph O. Wilmoth, Krista N. Langston, Mario Schootman, James P. Selig, Karen Hye-cheon Kim Yeary

**Affiliations:** a College of Medicine, University of Arkansas for Medical Sci10-1ences Northwest, 1125 N. College Avenue, Fayetteville, AR; b Department of Native Hawaiian Health, John A. Burns School of Medicine, University of Hawaii at Manoa, Honolulu, HI; c Center for Pacific Islander Health, University of Arkansas for Medical Sciences Northwest, Fayetteville; d Division of Biostatistics, Department of Preventive Medicine, College of Medicine, University of Tennessee Health Science Center, TN; e Arkansas Coalition of Marshallese, Springdale, AR; f Office of Community Health and Research, University of Arkansas for Medical Sciences Northwest, Fayetteville; g Department of Epidemiology and Biostatistics, College for Public Health and Social Justice, Saint Louis University, St Louis, MO; Department of Biostatistics; h College of Public Health, University of Arkansas for Medical Sciences, Little Rock, AR; i Department of Health Behavior and Health Education, University of Arkansas for Medical Sciences, Little Rock, AR.

**Keywords:** community-based participatory research, comparative effectiveness, diabetes prevention program, Marshallese, Pacific Islanders, randomized controlled trial

## Abstract

**Background:**

Pacific Islander populations, including Marshallese, face a disproportionately high burden of health disparities relative to the general population.

**Objectives:**

A community-based participatory research (CBPR) approach was utilized to engage Marshallese participants in a comparative effectiveness trial testing 2 Diabetes Prevention Program (DPP) interventions designed to reduce participant's weight, lower HbA1c, encourage healthy eating, and increase physical activity.

**Design:**

To compare the effectiveness of the faith-based (WORD) DPP to the culturally adapted (Pacific Culturally Adapted Diabetes Prevention Program [PILI]) DPP, a clustered randomized controlled trial (RCT) with 384 Marshallese participants will be implemented in 32 churches located in Arkansas, Kansas, Missouri, and Oklahoma. Churches will be randomly assigned to WORD DPP arm or to PILI DPP arm.

**Methods:**

WORD DPP focuses on connecting faith and health to attain a healthy weight, eat healthy, and be more physically active. In contrast, PILI DPP is a family and community focused DPP curriculum specifically adapted for implementation in Pacific Islander communities. PILI focuses on engaging social support networks to maintain a healthy weight, eat healthy, and be more physically active. All participants are assessed at baseline, immediate post intervention, and 12 months post intervention.

**Summary:**

Both interventions aim to cause weight loss through improving physical activity and healthy eating, with the goal of preventing the development of T2D. The clustered RCT will determine which intervention is most effective with the Marshallese population. The utilization of a CBPR approach that involves local stakeholders and engages faith-based institutions in Marshallese communities will increase the potential for success and sustainability. This study is registered at clinicaltrials.gov (NCT03270436).

## 1 Introduction

The Pacific Islander population is increasing rapidly in the United States (US). The most rapid growth has been in rural, Southern, and Midwestern states.^[1,2]^ Pacific Islanders are underrepresented in health research, as prior research has often aggregated data on Pacific Islanders and Asians into one racial category, which can obscure health disparities.^[3–8]^ While there has been limited research on Pacific Islanders, the available data indicate significant health disparities between Pacific Islanders and other racial/ethnic populations in the United States.^[9–18]^ For example, 23.7% of Pacific Islanders surveyed by the Centers for Disease Control and Prevention in 2010 reported a diagnosis of type 2 diabetes (T2D), which is higher than any other racial/ethnic group. Estimates of T2D in the Marshallese population range from 20% to 50%, compared to 8.3% for the US population and 4% worldwide.^[19–25]^ Pilot health screening research with the Marshallese community in northwest Arkansas (n = 401), documented extremely high incidence of T2D (38.4%) and prediabetes (32.6%) with 90% of Marshallese participants being overweight or obese.^[25]^

Overweight/obesity is the strongest modifiable risk factor for T2D,^[26]^ and even a weight loss of 5% to 10% of a person's body weight is clinically meaningful and reduces the risk for T2D.^[27–29]^ The diabetes prevention program (DPP) is an evidence-based program that has been shown to improve risk factors for T2D, including weight, eating habits, and physical activity, and to decrease the incidence of T2D by 58% across multiple settings in the general population and in multiple racial and ethnic populations.^[30,31]^ Recent systematic reviews noted the DPP has yet to be adequately tested in Pacific Islanders.^[31,32]^ However, a culturally adapted version of the DPP, Partnership for Improving Lifestyle Intervention (PILI) DPP, affected weight loss, albeit modest, among Native Hawaiians and other Pacific Islanders, mainly Chuukese, living in Hawaii.^[33,34]^ However, the effectiveness of the DPP has yet to be tested in Pacific Islander populations outside of the Pacific region and in contexts (e.g., churches) that could enhance its efficacy. Until the DPP's effectiveness can be confirmed in specific, disaggregated Pacific Islander populations, disparities in T2D are perpetuated.

This paper presents the protocol of a randomized controlled trial (RCT) designed to compare the effectiveness among Marshallese Pacific Islanders in Arkansas, Kansas, Missouri, and Oklahoma of 2 DPP programs: a faith-based DPP that was tested in African American Churches: the Wholeness, Oneness, Righteousness, Deliverance (WORD) DPP^[35,36]^ and PILI DPP. This trial has 2 objectives: to contribute to the evidence base for DPP efficacy among Pacific Islanders and to compare the effectiveness of 2 adapted DPPs—the WORD DPP adapted to include faith-based focus vs the PILI DPP adapted to include culture specific focus. The project is currently implementing the intervention, thus the subsequent description will reflect the study's current timeframe of March 2018; version 1. This study is approved by the University of Arkansas for Medical Sciences (UAMS) Institutional Review Board (IRB #207034). It is registered at clinicaltrials.g (NCT03270436).

## 2 Methods

### 2.1 Study setting

The study will be conducted in 32 Marshallese churches in Arkansas, Kansas, Missouri, and Oklahoma. Churches are a particularly appropriate setting for DPP intervention delivery in Marshallese groups. Prior needs assessments have shown that 96.51% of Marshallese report regular church attendance. Within the Marshallese culture, churches represent more than religious affiliation. They represent the primary social and hierarchical institution in the Marshallese community.^[37]^ Churches often represent clan and atoll affiliation for Marshallese migrants. Pastors and madam pastors have respected leadership roles within the community that are more akin to island chiefs than to American pastors.^[37]^

### 2.2 Community-based participatory research partnership

This study uses a community-based participatory research (CBPR) approach because of its demonstrated effectiveness in mitigating barriers perpetuated by historical trauma. The Marshallese community exhibits distrust in academic researchers that is a byproduct of their experience with US nuclear testing and the unethical scientific study of those Marshallese that were exposed to nuclear fallout.^[38]^ CBPR approaches provide a way to address the historical trauma experienced by the Marshallese by prioritizing research topics chosen by the community and includes the full participation of the community in all aspects of the research. By sharing power with community members, a CBPR approach builds trust between community members and academic researchers and is an effective way to engage minority participants in research.^[39–42]^

In 2012, a CBPR partnership was formed when UAMS began working with the Marshallese community in Arkansas, Kansas, Missouri, and Oklahoma using a participatory process to understand community assets and needs. This engagement process is described elsewhere.^[43–46]^ Through a 2-year engagement process using broad-based mixed methods and multiple focus groups, the CBPR team documented the Marshallese community's top priorities that included: T2D, obesity, and culturally appropriate care. The CBPR team has conducted several pilot studies related to T2D and obesity beliefs and behaviors,^[25,43,44,47]^ and a fully powered RCT to test a family model of diabetes self-management education.^[48,49]^ Throughout this work, community and academic partners have collaborated on planning, implementing, and disseminating research. The current study is based upon a direct request from Marshallese pastors to focus on the prevention of T2D and to provide health education in church settings. Table 1 outlines how core CBPR principles, as delineated by Israel et al,^[50]^ have been used in this comparative effectiveness RCT.

**Table 1:**
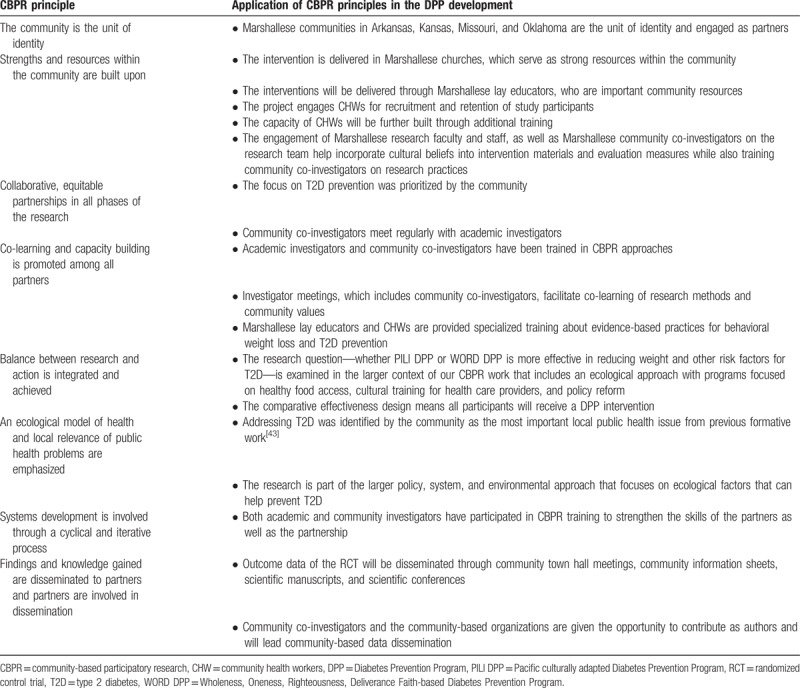
Application of community-based participatory research principles.^[49,50].^

### 2.3 Study aims

The primary aim of the RCT is to compare 2 DPP programs—the faith-based WORD DPP^[35,51]^ and the Pacific culturally adapted PILI DPP^[33,52]^—in the Marshallese population using a randomized cluster design. The primary outcome is percent body weight loss from baseline weight.

### 2.4 Theoretical framework

The project's conceptual framework is based on social cognitive theory (SCT)^[53]^ and social support and network models.^[54]^ Both recognize the interaction between individuals, their environment, and their behavior, with social support and network models focusing on the social environmental components (family, peers, and friends) of SCT. Both DPP interventions will use SCT-based behavioral strategies typically used in evidence-based behavioral weight control programs that will address the physical environment (e.g., stimulus control for weight loss), and the individual (e.g., self-monitoring) to cause behavior change. SCT-based strategies will be complemented with the engagement of social networks within Marshallese families and churches. Building upon these current social networks through implementing a group-based program within these social institutions (e.g., family, church) is hypothesized to facilitate changes in community or peer-group norms, and promote weight loss.

### 2.5 Study design and randomization

To assess the comparative effectiveness of WORD DPP and PILI DPP, a cluster RCT design will be used. A total of 384 participants who are overweight or obese will be recruited from 32 churches. Randomization will occur at the church level, with 1:1 assignment of 16 churches to each arm. Churches will be blocked (i.e., grouped into similar units) according to the geographic region and approximate number of adult church members. Using a computer generated algorithm for random assignment, half of the churches within each block will be assigned to WORD DPP arm and half will be assigned to PILI DPP arm of the study. Randomization will be conducted by a biostatistician who will have no interactions with potential participants and no supervisory role with study staff responsible for recruitment, consent, and intervention processes.

### 2.6 Study participant recruitment and screening

Participants will be recruited from Marshallese churches, clinics, community-based organizations, and social media in Arkansas, Kansas, Missouri, and Oklahoma. Church-based recruitment was specified by stakeholders as culturally appropriate and the community's preferred recruitment method. The study team has developed strong relationships with 52 Marshallese churches in the area, and the churches have agreed to work with study staff to inform participants about the study. Churches will distribute study information and will allow study staff to present information about the study to the entire congregation. To ensure that we reach those who do not attend church routinely, study information will also be provided through clinics, the respective states’ Departments of Health, community-based organizations, and social media.

The recruitment goal is 384 participants. During recruitment, Marshallese study staff will give presentations and distribute study information in English and Marshallese. Those who express interest will be invited to complete an eligibility screener questionnaire, which will ask about height, weight, date of birth, interest in participation, physical limitations, related co-morbidities, and questions regarding their ability to participate in physical activity. The Data Safety and Monitoring Committee, led by an endocrinologist and a Marshallese family practice physician, will review the results of the screener questionnaire before participants are allowed to enroll.

### 2.7 Participant inclusion and exclusion criteria

Marshallese adults (aged 18 and older) who have a body mass index (BMI) of ≥25 kg/m^2^ (i.e., classified as overweight or obese) will be eligible for the study. Persons who have a clinically significant medical condition likely to affect weight (e.g., cancer, human immunodeficiency virus/acquired immunodeficiency syndrome, etc.); are currently pregnant or breastfeeding an infant who is 6 months old or younger; or those who will not be able to complete the 6-month intervention will be excluded from participation.

All study information and materials will be provided in English and/or Marshallese according to each participant's preferences. The consent documents will be orally reviewed with eligible persons in a group and questions answered by Marshallese bilingual research staff. An opportunity will be provided to all potential participants for individual, private discussion of the study and the consent document before they sign the consent. A copy of the consent document will be given to the participant, and the informed consent process will be documented in the participant's research record. All members of the research team are trained and certified in participant consent procedures, the study protocol, and human subjects’ protection.

### 2.8 Data collection

Biometric and survey data will be collected at preintervention (baseline), postintervention (6 months after preintervention), and 12-month postintervention (i.e., 12 months from study initiation and 6 months after the first postintervention data collection). *Biometric measures* will include: measured weight, measured height, hemoglobin A1c (HbA1c), waist circumference, and blood pressure. Participants’ weight (without shoes) will be measured to the nearest 0.5 lb. (0.2 kg) using a calibrated scale. Height (without shoes) will measured to the nearest 0.25 inch using a stadiometer. Weight and height will be used to compute a continuous measure of BMI using the Quetelet Index (kg/m^2^).^[55]^ Systolic and diastolic blood pressure will be measured using a sphygmomanometer and stethoscope or digital blood pressure device with the participant seated and arm elevated. Finger stick blood collection will be used to test HbA1c using a Rapid A1c test kit and Siemens DCA Vantage Analyzer.^[56]^ The data collection will be completed by qualified, trained research staff. Bilingual study staff will be present to interpret for participants.

The survey instruments have been translated into Marshallese by a certified interpreter and tested by the study's Marshallese Community Advisory Board prior to implementation. The survey data collection includes 57 items adapted from valid and reliable scales, which will take participants approximately 20 minutes to complete.

Fruit and vegetable consumption will be measured using a questionnaire by Shannon et al.^[57]^ Sugar sweetened beverage consumption will be assessed with items from the Behavioral Risk Factor Surveillance System (BRFSS).^[58]^

Psychosocial variables including social support and self-efficacy for body weight, diet, and physical activity will be assessed. Weight locus of control is measured using the Weight Locus of Control scale.^[59]^ Family support for exercise and healthy diet will be measured by items from Gruber.^[60]^ Exercise self-efficacy will be measured using the self-efficacy for exercise and outcome expectations scale by Resnick et al.^[61]^ Self-efficacy scales for health-related diet and exercise behaviors will also be assessed using Clark et al's^[62]^ measure.

Other variables that will assessed will include food insecurity from National Health and Nutrition Examination Survey (NHANES),^[63]^ sleep quality, and quantity items are taken from the BRFSS,^[58]^ an item from Koenig and Büssing^[64]^ will be used to measure church attendance, and items assessing how often participants receive health messages at church were adapted from Ayers et al.^[65]^

In addition to biometric and survey data collection, participants will be invited to participate in a qualitative interview at 6-months postintervention to understand participants’ perceptions of the intervention and implementation process.

The study team will minimize missing data by using highly qualified staff to systematically collect data from all participants. The Research Electronic Data Capture (REDCap) platform will be used to monitor the occurrence of missing data during field collection.^[66]^ REDCap has a standard missing data report to facilitate the identification of missing data fields, which allows for continuous data quality monitoring so that missing data can be collected immediately. If a participant drops out, the study team will document why the drop out occurred. The study team will continue to collect information on all outcomes from participants, unless consent is withdrawn.

### 2.9 Remuneration

Participants are offered a $20 gift card as remuneration for their preintervention data collection; $30 gift card as remuneration for postintervention data collection; and $40 gift card as remuneration for their 12 month postintervention data collection. Participants will only receive gift cards for the data collection events they complete. Participants who participate in the qualitative interview will be given a $20 gift card.

### 2.10 Intervention description and delivery

Participants will either receive WORD DPP or PILI DPP, based upon random assignment. Marshallese stakeholders chose these 2 interventions because both interventions were developed through CBPR processes and both have shown effectiveness in other populations.^[33–35]^ Both interventions will be delivered at a church in a group setting. Both interventions’ core curricula emphasize self-monitoring, behavioral strategies for weight loss, decreasing caloric intake for weight loss, and increased moderate physical activity. Each group will be led by bilingual (Marshallese and English) DPP lay educators who each received at least 40 hours of DPP lifestyle coach training. Both interventions will offer materials in both English and Marshallese. Both interventions will offer makeup sessions for missed modules.

The WORD DPP curriculum is based on an adaptation of the DPP for rural African American communities of faith that utilized a CBPR approach.^[35]^ Preliminary efficacy data reports that African American participants of WORD DPP achieved 2.3% (standard deviation = 0.4) weight loss from baseline to 6-month follow-up. Participants attending at least half of the intervention sessions lost 3.7% (standard deviation = 0.6), and 21.4% of participants lost at least 5%. These results are comparable to the DPP studies in African Americans.^[67]^ For this study, community and academic partners revised The WORD curriculum that was originally designed for rural African American faith communities. The curriculum was changed to ensure relevance to Marshallese faith communities. The WORD DPP was not adapted for other aspects of Marshallese culture, such as the collective nature of the family. The WORD DPP includes 16 lessons delivered by trained community members over a 24-week period, with each lesson approximately 90 minutes in length. The first 8 lessons are to be delivered weekly, with the last 8 lessons to be delivered bi-weekly. Mirroring the core content of the DPP, the WORD DPP emphasizes reduced caloric intake, increased physical activity, and behavioral strategies for weight loss. Participants are also taught about the connection between faith and health and the importance of drawing from one's faith to make healthy changes.

The PILI DPP is a two-phase family and community-focused DPP curriculum that teaches participants to engage their family and community supports to achieve a healthy weight, eat healthy, and be physically active. The first phase is a culturally adapted DPP for Pacific Islanders that includes all of the original core DPP curriculum, with the addition of 2 topics on the economics of healthy eating (i.e., how to eat healthy within your budget) and talking with your doctor (i.e., communicating effectively with your healthcare provider).^[52]^ The 16 original DPP sessions were condensed into eight sessions, and 2 additional topics were included. The second phase is an extension of the basic DPP curriculum to include family and friends and to leverage existing community supports/resources to support long-term individual behavior changes. Specifically, participants are asked to elicit support from their friends and family, increase family activities around eating and being active, manage challenging social situations, effectively communicate one's healthy lifestyle goals, and identify and utilize community resources (e.g., parks and farmers markets). This second phase includes an additional 6 lessons. For this study, PILI DPP includes all 14 sessions delivered over a 24-week period, with each session lasting approximately 90 minutes. Participants will be encouraged to log their weight, physical activity, and nutrition on a daily basis. The PILI DPP has fewer contact hours than WORD DPP and is culturally adapted with examples relevant to Pacific Islanders.^[33,52]^ In prior studies, PILI has demonstrated significant weight loss (51% of participants reached ≥ 3% weight loss goal compared to 31.4% of those in a standard follow-up control group) and significant improvements in blood pressure and physical activity frequency and decreases in dietary fat.^[33,34]^

## 3 Data analysis

### 3.1 Power, sample size, and detectable effects

A cluster randomized design is employed to reduce contamination. Using the cluster randomized design with church as a cluster unit, we will recruit a sample size of 32 churches (16 clusters per arm with 12 individuals per cluster) for an overall sample of 384 individual participants. This number of churches and participants achieves 91% power to detect a difference of 2.5 kg (Standard Deviation = 7) (approximately 4% body weight loss) between the 2 groups’ mean body weight loss (effect size = 0.35) from pre- to postintervention assessment when the intra-cluster correlation is 0.01 using a linear model with a significance level of 0.05. We have 80% power to detect smaller effects if observed (effect size = 0.31).^[68–72]^ All power calculations were conducted with PASS12.^[73]^

### 3.2 Statistical analysis plan

All of the analyses will be performed with SAS/STATv14.1.^[74]^ Data will be examined for distributional normality and outliers prior to any analyses. Descriptive statistics will be generated for all variables of interest included in the analysis, overall and by intervention assignment. Univariate comparisons will consist of *t*-tests and ANOVA, and chi-square and other non-parametric tests, if needed, for continuous and categorical variables, respectively.

The distributional assumptions for outcome measures will be examined, which may prompt transformations if justified. The results of parametric and nonparametric univariate tests will also be compared (e.g., Wilcoxon test, Kolmogorov–Smirnov test, or Fisher's exact test, respectively) as a sensitivity analysis to examine the robustness of our findings. If distributional assumptions are not met, nonparametric tests will be applied.

Extent of randomization will be assessed by comparing intervention arms on baseline measures using *t*-test, chi-square test, ANOVA, and other appropriate tests. If imbalances are found, adjusting the between-group analyses for potential confounders will be considered.

Primary analyses will be intention-to-treat, without regard to intervention adherence. Multivariable linear ANCOVA regression models for continuous outcomes will be used to account for clustering effect within churches, to model and compare PILI DPP to WORD DPP. Using these models, treatment effects will be estimated and tested by comparing change from baseline in group-specific means at 6 and 12 months postintervention, conservatively adjusting for baseline differences and taking into account intra-cluster correlation by assuming compound symmetry covariance structure.

The main independent variable of interest is intervention assignment (PILI DPP vs WORD DPP) and the primary outcome is percent body weight loss from pre- to postintervention assessment. Measures of weight will be obtained at multiple points during the study (preintervention, postintervention, and 12-months postintervention), and changes from pre- to postintervention and preintervention to 12 months (6 months postinitiation) will be modeled in separate models. General linear and mixed ANCOVA regression models for continuous measures with clustering will be utilized, with the treatment effect estimated as the distance between the fitted group-specific means at the postintervention assessment, while adjusting for the fitted distance between them at baseline. In addition, conservative adjustments will be made within the model for demographic factors and other covariates listed above. In order to model the outcome at the 12-month time point, a similar model to the one described above will be applied with repeated measures, and incorporate intervention assignment, time, and their interaction effect, while adjusting for the same covariates as in the first model. This will allow the examination of weight change trajectories over time for both groups. Secondary outcome measures that are continuous in nature (e.g., hemoglobin A1c) will be modeled using the same approach as for the primary outcome (general linear mixed model). Secondary outcomes that are discrete (e.g., weight loss > 5% of baseline, meeting physical activity standard) will be modeled using generalized estimating equations (GEE) for repeated binary measures accounting for the correlation within churches. The effect of dosage (number of sessions completed) on outcome variables will be tracked and analyzed.

To account for missing data, imputation methods will be compared under several assumed missing data mechanisms, missing at random (MAR), and missing not at random (MNAR), in order to determine which underlying mechanism best fits the data. Given recent advances in handling missing data in longitudinal studies, several reasonable approaches are available that will be applied and compared. The results of the analysis will be compared using 3 approaches as a comprehensive sensitivity check: use a random effects model in SAS PROC MIXED (or weighted GEE for discrete outcomes) that makes use of all available data when assuming observations are MAR; perform multiple imputations (N = 25) in SAS PROC MI when assuming observations are MAR; and perform pattern-mixture model imputations in SAS PROC MI when assuming observations are MNAR. To address this potential source of inferential error, monotone regression based, multiple random imputations of the outcomes will be used. Demographic covariates and prior weight measurements that are available in this predictive model will be used. The analysis will then be carried out in multiple data sets, and the results will combined using standard methods in SAS PROC MIANALYZE to produce summary effect and standard error estimates that incorporate the imputation error.^[75,76]^

We will also determine if aspects of participants’ built environment as measured by Google StreetView^[77,78]^ and aspects of the food environment based on Business Analyst in ArcGIS influence the effectiveness of the intervention.^[79]^

### 3.3 Plans evaluate heterogeneity of treatment

Comparative effectiveness of the 2 DPPs will also be evaluated among subgroups to determine whether effectiveness varies for specific population segments. This will be done by testing 2-way interactions between intervention assignment and covariates of interest. These include sex, age, education, insurance status, and marital status. Bonferroni corrections will be applied to control *P*-values for multiple comparisons. For these exploratory comparisons, all relevant subgroup outcomes will be analyzed and reported.

### 3.4 Data safety and monitoring

This study poses minimal risk. The Data Safety Monitoring Committee is composed of a Marshallese family physician, an endocrinologist, and a health educator from the Marshallese community. The Data Safety Monitoring Committee reviews for participant eligibility as well as adverse events.

### 3.5 Data sharing plan

The study team will construct a complete, cleaned, and de-identified copy of the final dataset used in conducting the final analyses. This data set will be made available to other researchers. Researchers interested in accessing data will be asked to submit a letter of intent (LOI) that describes their proposed research, the types of data required, and a demonstration of adequate expertise to conduct the proposed research. The LOI also requires information regarding resources available for the proposal: funding source (s), equipment, and technical support. Each LOI will be reviewed by a committee at UAMS that includes the principal investigator or a co-investigator and members of the Marshallese community. Researchers will sign data use agreement to ensure proper handling of the data.

## 4 Dissemination plan

Dissemination is crucial to achieving research impact and benefits for stakeholders. Academic dissemination will include peer-reviewed journals and academic conferences. Manuscripts will adhere to CONSORT reporting guidelines for cluster randomized trials.^[80]^ Marshallese community partners will be invited to co-author and co-present research with the research team, and are co-authors of this paper. In addition, when conducting research to address health disparities, there is an ethical responsibility to disseminate findings back to participants and community members. The Agency for Healthcare Research and Quality's Dissemination Planning Tool^[81]^ will be used as the framework for dissemination.

### 4.1 Dissemination to study participants

Results will be returned to participants first. Each participant will be mailed or e-mailed a one-page summary of results that is formatted as an infographic. The infographic will be presented in English on one side and in Marshallese on the other side. The infographic will use plain language suitable for all audiences, including those with low health literacy. The infographic will use culturally relevant pictures/examples. This type of infographic is preferred by Marshallese stakeholders and has been successfully used during our prior studies to provide results to participants. The study team has incorporated careful measures to protect the participants involved in the study. Aggregate results will be disseminated and no protected health information (PHI) will be shared. In addition, participants will be invited to the town hall meeting discussed below.

### 4.2 Dissemination to the broader marshallese/pacific islander community

A CBPR approach values co-learning, transparency, reciprocity, and partnerships; therefore, it is important to disseminate regular study updates as well as final results to the broader community. Marshallese stakeholders have expressed a preference for periodic updates on progress throughout the study. Study updates and final results will be disseminated during biannual town hall meetings that will be hosted by the study's community-based partner (Arkansas Coalition of Marshallese) and facilitated by the community co-investigators. At these meetings, the research team (including Marshallese study staff and community co-investigators) will present updates on enrollment and retention. When the study concludes, the results and lessons learned will be presented using an easy to understand infographic, as described above. Marshallese stakeholders use social media as a primary means of communication within the community. Therefore, recruitment information, study updates, and dissemination of final results will be provided through Facebook and other websites. Only aggregate information without PHI will be shared.

## Acknowledgments

The community engagement efforts were supported by the UAMS Translational Research Institute funding through the United States National Institutes of Health (NIH) National Center for Research Resources and National Center for Advancing Translational Sciences (UL1TR000039). The research to test the adapted curriculum was partially funded through a Patient-Centered Outcomes Research Institute (PCORI) Award AD-1603-34602 and by the Wal-Mart Foundation. The content presented in this publication is solely the responsibility of the authors and does not necessarily represent the views of NIH, PCORI, or the Wal-Mart Foundation. The project is made possible because of the existing community-based participatory research partnership with the Marshallese Consulate General in Springdale, Arkansas and the Arkansas Coalition of Marshallese.

## Author contributions

**Conceptualization:** Pearl McElfish, Joseph Keawe‘aimoku Kaholokula, Karen Hye-cheon Kim Yeary.

**Funding acquisition:** Pearl McElfish.

**Methodology:** Christopher R. Long, Zoran Bursacv, Mario Schootman, James Selig.

**Project administration:** Williamina Bing, Ralph Wilmoth, Krista Langston.

**Software:** Zoran Bursac.

**Validation:** Zoran Bursac.

**Writing – original draft:** Pearl McElfish, Karen Hye-cheon Kim Yeary.

**Writing – review & editing:** Christopher R. Long, Joseph Keawe‘aimoku Kaholokula, Nia Aitaoto, Lucy Capelle, Melisa Laelan, Williamina Bing, Sheldon Riklon, Brett Rowland, Britni L. Ayers, Ralph Wilmoth, Mario Schootman, James Selig.
